# “I know it’s not good, but you want to perform at 100%”: cognitive-affective responses to ecological sustainability and performance in sport competitions

**DOI:** 10.3389/fpsyg.2026.1880290

**Published:** 2026-08-21

**Authors:** Christian Kraft, Rabea Bödding, Maximilian Gottwill, Christian Zierke, Agnieszka Paruzel, Günter W. Maier, Pamela Wicker

**Affiliations:** 1 Department of Sports Science, Bielefeld University, Bielefeld, Germany; 2 Department of Psychology, Bielefeld University, Bielefeld, Germany

**Keywords:** athletes, climate change, emotions, environmental sustainability, sport performance

## Abstract

**Introduction:**

Competitive sport is increasingly affected by environmental challenges. Athletes compete in performance-oriented systems that often constrain pro-environmental action. Navigating ecological sustainability-related cognition and affect may therefore impose additional mental load on athletes and require self-regulation, potentially competing with performance-relevant cognitive and emotional resources, consistent with resource-based models of self-control. However, little is known about how athletes experience ecological sustainability within their competitive sport and how they perceive its impact on performance. This qualitative study examined how competitive athletes cognitively and affectively engage with ecological sustainability and how these experiences are perceived in relation to their sporting performance.

**Methods:**

Semi-structured interviews were conducted with nine athletes from different sports and competitive levels. Data were analyzed using structured deductive qualitative content analysis.

**Results:**

Athletes expressed strong pro-environmental attitudes but experienced a pronounced and recurring value-action gap driven by structural constraints such as competition schedules, travel demands, and sponsorship obligations. Ecological sustainability-related cognition and affect (e.g., guilt, frustration, sadness) were salient across sports but were actively regulated, suppressed, or compartmentalized in performance contexts to maintain focus and competitive readiness. While athletes rarely reported immediate performance impairments, sustainability-related concerns contributed to indirect performance-relevant strain through travel fatigue, disrupted preparation, and cumulative mental load affecting recovery and well-being. Engagement in sustainability initiatives emerged as a central coping strategy and was often experienced as empowering; sport itself served as an important coping context.

**Discussion:**

Overall, ecological sustainability functioned both as a psychological burden and a motivational resource in competitive sport. While athletes expressed strong motivation to engage with sustainability, their ability to act consistently with these values was often constrained by both the structures of competitive sport and individual decisions.

## Graphical abstract

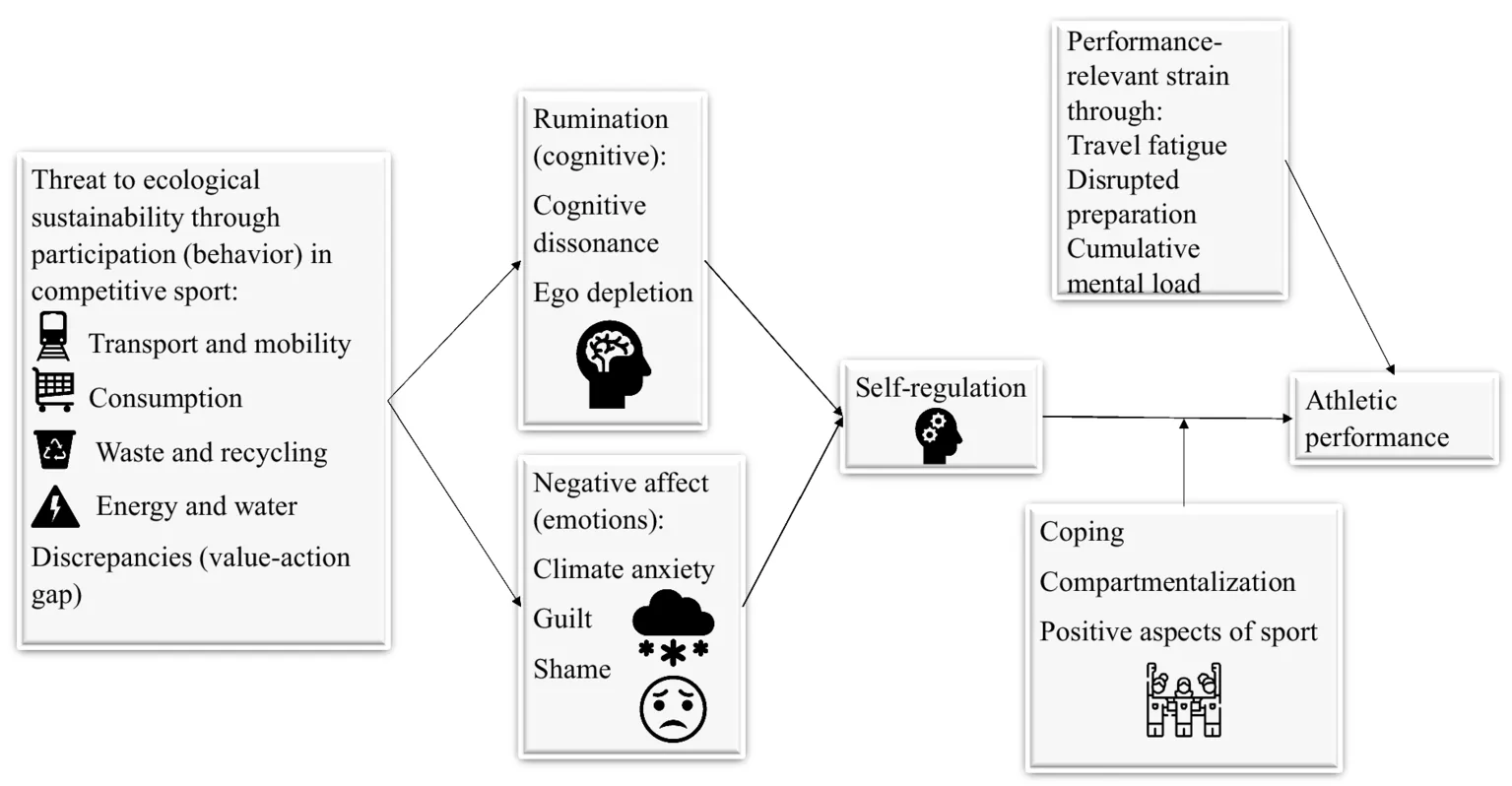

## 1 Introduction

Climate change poses an ongoing risk to nearly every aspect of human life, with even more severe impacts anticipated in the mid-21st century (IPCC, 2018; Scott et al., 2018). Sport participants (particularly in outdoor sports) are at heightened risk from these changes (Bernard et al., 2024; Schneider and Mücke, 2024). Competitive athletes are at particular risk because they must adhere to externally imposed competition schedules that are often not adjusted for climate change and changing weather conditions (Mallen et al., 2025; Orr et al., 2022).

A growing body of literature outlines the manifold risks climate change poses to athletes at all levels of competition (Orr et al., 2022; Schneider and Mücke, 2024). For example, ozone layer depletion increases UV radiation and skin cancer risk for outdoor athletes (Schneider and Mücke, 2024). Increasing air and water temperatures impose further risk on athletes, such as heat illness, heat stroke, and bacterial infections from worsened water quality (Grantham et al., 2010; Schneider and Mücke, 2024). In addition, warmer winters increase the risk of injuries and accidents due to poorer snow quality (Derman et al., 2018; Schneider et al., 2025). Recent numbers show that over 75% of Olympic athletes are already experiencing negative effects of climate change on their health and performance (World Athletics, 2023), with this impact being even more severe in winter sports (IBU, 2026).

In response to such challenges, athletes have begun voicing their concerns and engaging in ecological sustainability advocacy (e.g., United Nations Framework for Climate Change, n.d.; Protect Our Winters, n.d.). Athlete-led environmental initiatives are growing, with increasing demands for more ecologically sustainable calendars, venues, and event logistics. However, athletes operate within rigid, externally imposed structures that restrict behavioral autonomy. Competition schedules, (inter)national travel, sponsorship obligations, and equipment standards could conflict with pro-environmental intentions. This limited autonomy could lead to tensions between athletes’ individual environmental attitudes and values and the sport-related behavioral demands (Wågan and Wilson, 2025). At the same time, athletes are not merely affected by these structures but also participate in sport systems that contribute to environmental pressures through travel, consumption, and resource use. Athletes may, therefore, experience tensions arising not only from being affected by climate change but also from their own involvement in environmentally consequential practices.

This tension may persist when athletes hold pro-environmental attitudes but are unable to behave accordingly (Stoknes, 2015). In competitive sport, structural demands such as travel, equipment use, or scheduling constraints may create a mismatch between environmental values and required behaviors, potentially leading to psychological strain. To better understand these tensions, cognitive dissonance theory (Festinger, 1957) and resource-based models of self-control (Baumeister et al., 2007) suggest that value-behavior conflicts may elicit psychological strain, the regulation of which draws on limited mental resources and may become relevant for performance.

Despite this relevance, there is a lack of research examining how athletes experience ecological sustainability cognitively and affectively, particularly in relation to their performance in sport competitions. Moreover, understanding these experiences is not only relevant for athletes’ performance, but is also societally important. Athletes are not only increasingly affected by climate change but are also embedded within sport systems that contribute to environmental pressures (Orr et al., 2022; World Athletics, 2023). How athletes perceive and navigate sustainability-related tensions may, therefore, provide insights into how the consequences of environmental practices are maintained, challenged, or transformed within competitive sport. Moreover, athletes often occupy highly visible public roles and may contribute to public sustainability discourses through advocacy and communication efforts (Trendafilova et al., 2014). Understanding their experiences may inform discussions about sport’s potential contribution to broader sustainability transitions. Having said this, athletes from different sport contexts may experience ecological sustainability in different ways. Sports vary in their dependence on natural environments, exposure to climate change impacts, travel demands, and the extent to which environmental attitudes are integrated into athletes’ sport identities (Orr et al., 2022; Jansen et al., 2024). Rather than comparing sport disciplines, the present study examines how athletes experience ecological sustainability-related cognition (e.g., thoughts about emissions, mobility, or equipment use) and affect (e.g., worry, guilt, helplessness) within their sport and how these are perceived and negotiated in relation to sporting performance. Specifically, the study addresses the following research questions^1^: (1) How do competitive athletes cognitively and affectively engage with ecological sustainability within their sport? And (2) How do competitive athletes perceive the relevance of ecological sustainability-related cognition and affect for their sporting performance?

By answering these questions, this work makes two contributions. First, it integrates previously fragmented research by jointly examining two links that have been studied separately: The relationship between sustainability-related demands in sport and athletes’ cognitive and affective processes (Bernard et al., 2024; Clayton, 2020), and the relationship between cognitive and affective processes and sporting performance (Chen et al., 2025; Wagstaff, 2014). Second, by adopting a qualitative approach, it offers an in-depth analysis of relevant dimensions of these processes. The findings offer implications for athletes, coaches, sport psychologists, as well as event managers and organizers.

## 2 Theoretical background

The present study draws on cognitive dissonance theory (Festinger, 1957) and resource-based models of self-control (Baumeister et al., 2007) to explain how ecological sustainability-related demands may give rise to performance-relevant psychological processes in competitive sport. According to cognitive dissonance theory (Festinger, 1957; Hinojosa et al., 2017), the confrontation with a mismatch between personal values and actual behavior can elicit a state of psychological arousal, which may develop into psychological strain if not resolved. In the context of competitive sports, athletes may hold pro-environmental attitudes but feel compelled to act against these values due to structural demands such as frequent travel, equipment use, or scheduling constraints. As a result, unresolved dissonance may give rise to persistent rumination and negative emotional responses such as guilt, shame, or anxiety (Harmon-Jones et al., 2025; Lavergne and Pelletier, 2015). Against this background, cognitive and affective responses are central to understand how athletes experience ecological sustainability-related tensions in sport.

In general, cognition refers to mental processes of knowing and processing information (Bender and Beller, 2020). Here, ecological sustainability-related cognition is defined as thought processes related to the environmental aspects of participating in competitive sport, including reflections on emissions from travel, the environmental impact of sports equipment and apparel, and resource use at venues (e.g., energy or water). Such cognitions can be broadly categorized along four behavioral domains: Transport and mobility; consumption; waste and recycling; and energy and water saving (Diekmann and Preisendörfer, 2003).

Affect refers to bodily feelings, moods, and sensations that can shift in intensity (Gregg and Seigworth, 2010). In the present study, ecological sustainability-related affect refers to the emotional reactions athletes experience in relation to ecological concerns such as worry, sadness, frustration, or climate anxiety (i.e., an enduring emotional response to the climate crisis; (Clayton, 2020; Lutz et al., 2023)). These emotional responses may be directed toward the athlete’s own behavior or broader environmental changes that affect sport participation.

From a transactional perspective on stress and emotion (Lazarus and Folkman, 1984), emotional responses arise from individuals’ cognitive appraisals of environmental demands in relation to personal goals and coping resources, meaning cognition and affect are intertwined (Dreisbach, 2022). Applied to competitive sport, ecological sustainability-related affect can thus be understood as resulting from athletes’ appraisals of environmentally harmful practices (e.g., travel, resource use) as stressful or morally conflicting to personally valued goals (e.g., acting ecologically sustainable).

Importantly, both cognition and affect are known to influence behavior and performance in sport (Birrer et al., 2012; Lopes, 2024). Following the strength model of self-control (Baumeister et al., 2007), engaging in complex psychological regulation (e.g., suppressing environmental guilt while preparing for competition) draws on limited mental resources (Baumeister et al., 1994; Englert, 2016). The more athletes are preoccupied with ecological sustainability-related concerns, the greater the risk of cognitive overload. This overload potentially leads to diminished focus or impaired recovery due to ego depletion (Baumeister et al., 1994; Englert, 2016), that is, a temporary reduction in self-regulatory capacity following prior mental effort. This can reduce athletes’ capacity to employ performance-enhancing strategies. Although athletes often display coping mechanisms to protect performance (Chen et al., 2025), the cumulative load of unresolved ecological sustainability-related cognition and affect may still undermine performance (Englert, 2016). Several studies demonstrate ego depletion effects across exercise disciplines and tasks (Wagstaff, 2014; Dorris et al., 2012; Englert and Wolff, 2015).

**Figure 1:**
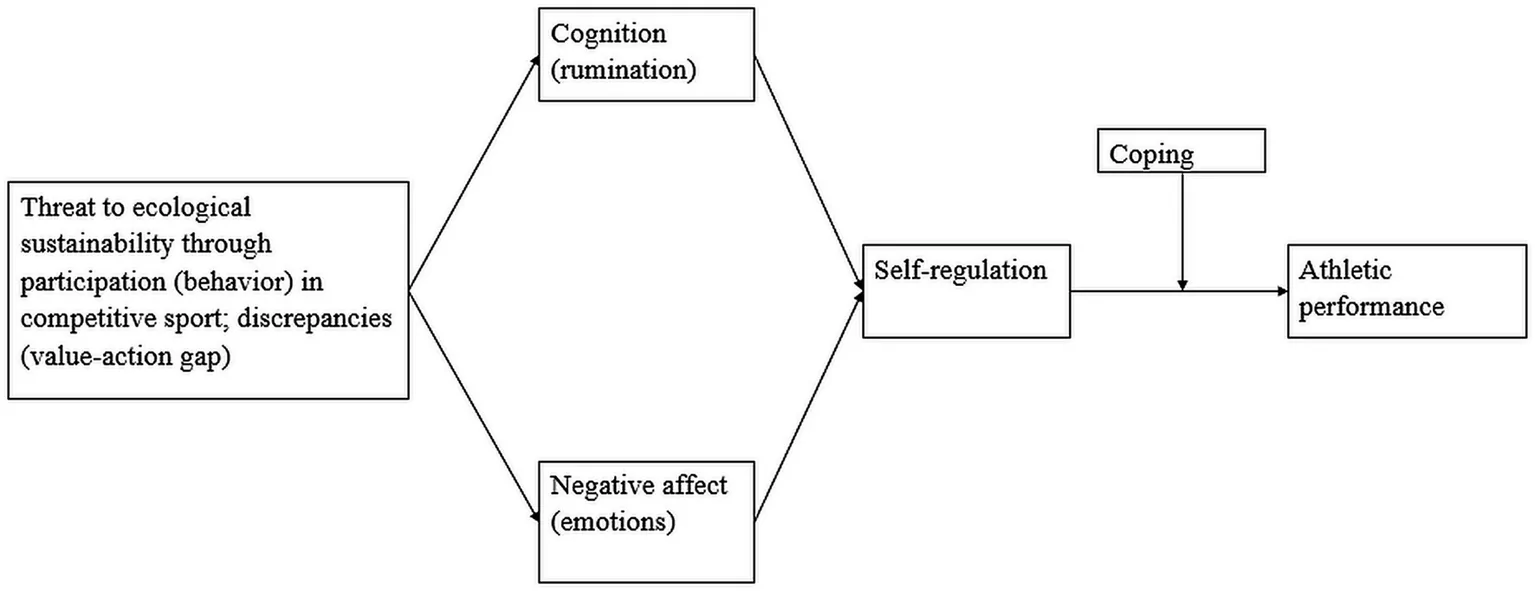
Theoretical process model of a possible psychological pathway between athletes’ ecological sustainability-related cognition and affect and athletic performance.

Building on these theoretical considerations, a process model was developed to guide the present study (Figure 1) and inform the subsequent qualitative analysis. The model outlines a potential psychological pathway through which ecological sustainability-related demands in sport may influence athletic performance. Specifically, awareness of sustainability-related impacts of sport participation (e.g., transport and mobility, consumption, or resource use; Diekmann and Preisendörfer, 2003) may elicit cognitive (e.g., rumination) and affective (e.g., negative emotions) responses. These responses may create psychological strain that requires self-regulation. Drawing on the strength model of self-control (Baumeister et al., 2007; Thomas et al., 2013), such regulation consumes limited mental resources and may impair performance through mechanisms such as ego depletion or mental fatigue. At the same time, coping strategies (e.g., rationalization or compartmentalization; Englert, 2016) may buffer or amplify these effects.

## 3 Methods

### 3.1 Participants

Participants were recruited via the communication channels of the Department of Sports Science, including mailing lists of faculty and students as well as social media channels. Of interest were athletes who actively compete at any level with a minimum age of 18 years. The study advertisement informed potential participants that the interviews would focus on thoughts and emotions related to ecological sustainability in sport. Thirteen individuals stated interest in the study, of which nine participated (I1-I9). Interviews were conducted until thematic saturation was reached, that is, no additional aspects relevant to the research questions emerged during the interviews. One additional interview was conducted to confirm that no new aspects relevant to the research questions emerged. Table 1 summarizes the characteristics of the interviewees. The interviewees comprised 6 women and 3 men (age range: 19–34 years, *M* = 24.56). The athletes have participated in sport competitions between 4 years and 16 years (*M* = 11.44). The competitive levels ranged from mass sport athletes to international-level athletes. The minimum number of competitions (games, races, events) in 2025 was 7, with 20 being the maximum (*M* = 12.1). Perceived sport-specific risk due to climate change was rated on a 5-point Likert scale (from 1 = *not affected at all* to 5 = *strongly affected*) with an average of *M* = 2.89. The practiced sports included year-round (e.g., football, tennis) and seasonal disciplines (e.g., triathlon, biathlon).

**Table 1:** Overview of characteristics of study participants.

Interview	Sport	Age	Gender	# of games/race days/competitions per year	Competition level	Years competing	Risk perception	Interview duration
1	Tennis	24	Male	7 (tournaments)	District/regional level	10 years	3	29:56 min
2	Road cycling	28	Male	20 (race days)	Elite-amateur	16 years	3	15:01 min
3	Track and Field	24	Female	8 (competitions)	Regional level	14 years	2	21:26 min
4	Triathlon	34	Female	8 (races)	2. Bundesliga (nationwide)	7 years	4	24:35 min
5	Football	23	Female	20 games	Regional league (north)	16 years	3	24:07 min
6	Triathlon	20	Male	7 (races)	2. Bundesliga (nationwide)	4 years	4	19:51 min
7	Speed Climbing	19	Female	6 (competitions)	National climbing team (European championships)	7 years	1	21:46 min
8	Long-distance running	25	Female	16 (races)	German and federal state championships	7 years	2	19:32 min
9	Biathlon	24	Female	11 (races)	IBU World Cup (Olympic Games)	10 years	4	25:08 min

Risk perception was assessed by asking athletes to rate the question: “To what extent do you think your sport is affected or at risk due to climate change or ecological changes?” on a scale from 1 = not affected at all to 5 = strongly affected.

### 3.2 Procedure

The interviews were conducted online via a video-conference tool by the lead author. They were held between December 2025 and January 2026 and lasted 15–30 min (*M* = 22:22 min). Despite their relatively short duration, the interviews were designed to be focused and thematically dense, enabling participants to reflect on concrete experiences within a clearly defined performance context. Eight interviews were conducted in German and one in English. Different strategies were implemented to decrease social desirability. First, participants self-selected the date and time of the interview, as well as the interview location (private spaces). Second, the purpose of the study, as well as all relevant information regarding data collection and usage (e.g., content, aim of the study, anonymity, voluntary participation, and recording) were explained in advance. To participate in the interviews, the interviewees had to give their written consent. The study received ethical approval from the University’s Ethics Review Board. The study (research questions, method, and data extraction procedures) was pre-registered at the repository of the open science framework (OSF).^2^

**Figure 2:**
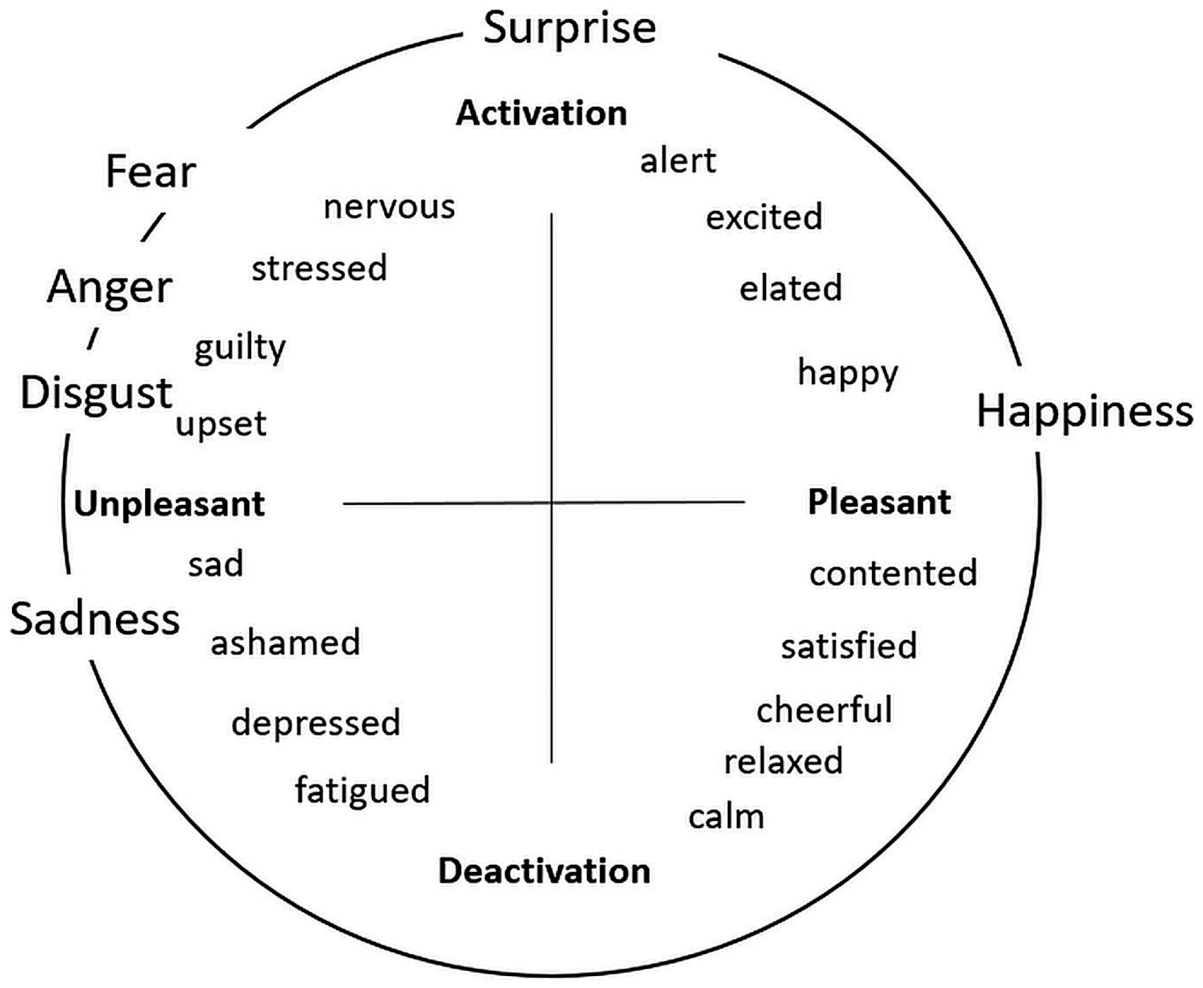
Circumplex model (Russell, 1980). Adapted from Pătruţ and Spatariu (Pătruţ and Spatariu, 2016).

Based on the theoretical model outlined in the introduction (Figure 1), an interview guide was developed by the lead author. The interview guide was refined through multiple rounds of discussion and feedback among the co-authors. All semi-structured interviews followed the same guide with 10 key questions and is provided in the Supplementary material. The interview started with general questions regarding the athlete and their respective sport. Afterwards, questions about individuals’ environmental perspectives in general and in their sport were posed. Then, athletes were asked about ecological sustainability-related cognition (thoughts) and affect (emotions) they encounter in their sport. Since naming and differentiating affective experiences can be challenging in semi-structured interviews, the circumplex model of affect (Figure 2; Russell, 1980) was presented to the athletes as a visual aid. The model was selected because it provides a comprehensive and non-prescriptive representation of affective experiences, enabling participants to describe emotions beyond predefined categories while facilitating emotion differentiation during the interview. Athletes were also asked about the influence of thoughts and emotions on their performance and possible coping mechanisms.

### 3.3 Empirical analysis

All interviews were audio-recorded and transcribed verbatim. Interviews were first reviewed in MAXQDA, a computer-assisted qualitative data analysis software. Subsequently, a structured, theory-driven deductive qualitative content analysis was conducted. The coding followed the systematic procedure outlined by Mayring (2021). Category development was guided by the theoretical process model (Figure 1) and the literature underpinning the interview guide. Thus, the main coding categories (e.g., ecological sustainability-related cognition, affect, performance relevance, and coping) were deductively derived from the theoretical framework and the corresponding interview domains, which were informed by previous research on cognition, affect, rumination, coping, and sport performance (Clayton, 2020; Chen et al., 2025; Wagstaff, 2014; Lazarus and Folkman, 1984; Baumeister et al., 1994; Englert, 2016). The analysis remained primarily deductive throughout. Repeated engagement with the interview material served to refine coding definitions, anchor examples, and coding rules rather than to develop new categories. The lead author familiarized himself with the data by repeatedly reading the transcripts. During this process, categories were refined, and coding definitions, anchor examples, and coding rules were developed. For quality assurance, interviews were then double-coded by two co-authors using the previously developed coding guide. Coding responsibilities were distributed across the dataset, with one coder responsible for Interviews 1–5 and another for Interviews 6–9. Interrater reliability was assessed using Cohen’s kappa (Brennan and Prediger, 1981), yielding substantial agreement (*κ* = 0.73 for Interviews 1–5; *κ* = 0.69 for Interviews 6–9). Given the interpretative nature of qualitative analysis, some discrepancies between coders were expected. Intercoder reliability was used as an indicator of the consistency and transparency of the coding process rather than as a requirement for complete agreement (O’Connor and Joffe, 2020). For reporting, illustrative quotes were extracted and translated into English.

## 4 Results

The presentation of results follows the previously developed theoretical process model, which incorporates the categories derived from the qualitative analysis.

### 4.1 Value-action gap in competitive sport

All interviewed athletes reported having positive pro-environmental values and attitudes. Participants emphasized the importance of environmental protection and ecological sustainability in general, as illustrated by statements such as: “*I would say that I do place a certain value on environmental protection, definitely on environmental protection, but also more broadly on sustainability and dealing with climate-related issues*” (I4). Often these values come from their family background and how their parents raised them: “*In general, I would say that I am quite environmentally conscious, mainly because this is how I was raised by my parents and my social environment*” (I5) but still these attitudes develop over time as well: “*And throughout the years, I’ve tried to live more and more sustainably myself*” (I9).

However, despite this value orientation, athletes described tensions between their attitudes and the practical demands of competitive sport: “*I would get on a plane again because it was simply more convenient … I know it’s not good [for the environment], but you want to perform at 100%, and that’s hard if you spend ten hours on a train*” (I7). In many cases, participants reported difficulties translating their environmental values into consistent pro-environmental behavior in their sport, pointing to a discrepancy between values and actions: “*In general, I would say that this is a very typical case for me: The environmental awareness is there, knowing how one should act or what would be the ‘right’ way to behave. (…) But on the other hand, when it comes to actually putting this into practice, it often does not happen*” (I1).

Within the four categories of pro-environmental behavior, which the athletes observe in their daily practices, transport and mobility play the most important role: “*The number one thing is definitely the travel. Because like this season, I do have to take three planes, or go by plane three times to go to competitions*” (I9). This is not an individual perspective but counts for athletes in general: “*It’s one of the biggest concerns amongst athletes… it should be much less travel for athletes*” (I9) and across sports: “*You do have to say that, to practice the sport itself, you travel by car a lot, and especially when you compete internationally*” (I4).

Consumption in competitive sport constituted another central area of perceived environmental tension, particularly regarding sport gear and equipment. Athletes described receiving large amounts of new clothing each season through clubs or sponsors, often regardless of actual need. One participant noted that they accumulate far more sport clothing than they can use, even though old equipment is still functional after one season: “*We get new clothing every season, and that’s an extreme amount of clothing… You always get new things, and after one season the items aren’t even broken*” (I5). At the same time, athletes acknowledged discrepancies between their private consumption ideals and their sport-related consumption practices. While some reported being mindful consumers in everyday life, they admitted that they tended to purchase new sport apparel more frequently than necessary: “*When it comes to sportswear in general, I would not say I’m a role model. I spend a lot of time in sports clothing, and I do enjoy placing a new order. I could definitely be more sustainable*” (I3). Second-hand options were generally perceived as less feasible in the sport context, particularly for sponsored athletes who are contractually obliged to wear specific brands. One athlete explained: “*Otherwise, I do wear second-hand clothes, but at that point I do not really hold back, because I have the privilege of being sponsored by a brand and receiving the clothes from them*” (I8). Conversely, athletes without sponsorship emphasized financial constraints as a driver of more ecological sustainability. For instance, one participant reported deliberately purchasing used equipment both for economic reasons and to avoid unnecessary new purchases: “*I think I have never bought a new bike, neither my road bike nor my triathlon bike. They are all second-hand bikes. One reason is clearly financial, but another reason is that they are still great*” (I4). Overall, these statements indicate a pronounced environmental value-action gap: Although athletes expressed strong pro-environmental attitudes, structural conditions within competitive sport (e.g., sponsorship contracts, performance norms, team provisions) constrained their ability to enact these values in everyday sport behavior.

Trash and recycling were mentioned less frequently but emerged as relevant aspects of ecological sustainability. Some athletes described taking responsibility for their own waste during competitions, especially in nature-based events: “*Everything I produce as waste, I take with me again*” (I8). However, participants also emphasized structural constraints within organized sport that generate large amounts of unavoidable waste. For example, team catering and packaging were described as highly wasteful: “*Everything is still wrapped in plastic… It all gets thrown away, there’s just an enormous amount of waste generated*” (I5). Similarly, medical practices such as taping were perceived as ecologically problematic but necessary for performance and injury prevention: “*Physio produces an enormous amount of waste… but that’s medical, you cannot really avoid it*” (I5). A few athletes referred to institutional sustainability initiatives, such as attempts to improve recycling at venues or projects aimed at recycling sport materials like carbon fiber: “*They also have a project about carbon recycling because carbon is a material that’s widely used in a lot of sports. … But currently they are not being recycled, which is a problem*” (I9). Overall, while athletes expressed awareness and concern regarding waste and recycling, their statements illustrate a recurring discrepancy between personal environmental values and the waste-intensive practices embedded in competitive sport structures.

Energy and water use were mentioned by a few athletes, who referred to concrete ecological sustainability measures in this domain, mainly at the level of sport facilities and event organization rather than individual behavior. For instance, one tennis player noted efforts to reduce energy consumption in indoor facilities: “*The halls are never set to maximum brightness… they usually run at around 70%*” (I1). In winter sports, athletes pointed to energy- and water-intensive technical measures that are framed as necessary for competition continuity, such as artificial snow production: “*[Organisation 4] has some project about snow farming, where they are trying to find the most sustainable way to produce snow, artificial snow*” (I9). Overall, while energy- and water-saving measures were occasionally recognized, they appeared less salient in athletes’ everyday reflections and were often perceived as organizational responsibilities rather than areas of individual agency within competitive sport contexts.

### 4.2 Rumination (cognitive)

Building on athletes’ pro-environmental attitudes and the discrepancies arising from constrained autonomy in competitive sport, two interrelated response pathways can be distinguished: Cognitive and affective. Starting with cognitive responses, athletes reported diverse ecological sustainability-related thoughts that varied in salience, duration, and perceived relevance, sometimes leading to rumination about perceived discrepancies in their environmental practices. For many athletes, ecological sustainability-related cognition was described as situational, emerging primarily in specific moments such as during travel or visible resource use. For instance, one tennis player reported that thoughts about water use occurred briefly when courts were irrigated during breaks, but quickly faded again: “*Then the thought comes up for a moment… that’s quite a lot of water being used there… These are really just very short thoughts that last maybe three seconds*” (I1). Similarly, several athletes emphasized that ecological sustainability rarely occupied their minds during training or competition, as performance-related demands dominated their attention: “*Honestly, almost not at all. When you go to competitions, you do not really think about it*” (I3).

Despite high self-reported environmental awareness, athletes reported cognitive discrepancies between values and behavior, particularly in relation to transport and mobility. One cyclist reflected on the tension between personal environmental values and the realities of competitive sport:

*It does conflict at times. On the one hand, I have this green mindset, but on the other hand, I’m part of a very non-ecological lifestyle… The sport is anything but sustainable when you drive across the country five times a year for races* (I2).

In some cases, such discrepancies triggered more persistent cognitive engagement and rumination. Athletes questioned whether certain practices were necessary or could be organized differently: “*Sometimes I ask myself whether it really has to be this way. Couldn’t it also be done differently?*” (I4).

However, several athletes reported deliberately limiting or suppressing ecological sustainability-related thoughts to protect their focus and performance, as these were perceived as distracting: “*It goes through my head briefly, but then other competition-related thoughts take up much more space. I do not give it too much room because I feel it would distract me*” (I8).

Taken together, athletes’ cognitive responses ranged from momentary reflections to more pronounced awareness of value-action discrepancies. Yet, these cognitions were often subordinated to performance demands, with athletes actively regulating ecological sustainability-related thoughts.

### 4.3 Negative affect (emotions)

Cognitive and affective responses were closely intertwined. Athletes’ ecological sustainability-related thoughts often elicited emotional reactions, while emotions, in turn, shaped how strongly athletes engaged cognitively with ecological issues. Overall, athletes reported a broad spectrum of emotions, with negative affect being more salient than positive ones. However, the emotional salience of ecological sustainability-related issues varied between individuals. Some athletes initially reported difficulty articulating emotions related to ecological sustainability: “*Honestly, I’ve never really thought about it in that much detail*” (I1).

When affects were reported, they were often low-intensity but recurrent, including discomfort, sadness, frustration, and guilt. For instance, athletes described feeling uncomfortable or surprised when confronted with visible resource use: “*The feeling that fits best is ‘unpleasant’, maybe mixed with being surprised. When you suddenly realize how much water is being used during a break, that feels uncomfortable*” (I1). Sadness and disappointment were frequently expressed in relation to broader societal and environmental developments: “*In general, it makes me sad how most people deal with sustainability and the environment*” (I2). Several athletes articulated moral emotions, particularly guilt and shame, arising from their perceived involvement in environmentally harmful practices through sport participation: “*I would say annoyed…but also 100% guilty. I’m part of the system, I use the materials, I travel, I benefit from the material, so I cannot take myself out of that responsibility*” (I5) or: “*Sometimes I feel a bit ashamed, especially when I hear how much people travel for competitions. You ask yourself: Does this really have to be?”* (I4).

In some cases, athletes also reported frustration or anger, particularly when ecological sustainability measures were perceived to interfere with performance or recovery (e.g., long train travel instead of flights): “*When we were told we had to take the train instead of flying, we were quite frustrated, because we knew our performance the next day would not be optimal*” (I7).

In addition to discrete emotions such as guilt or frustration, some athletes reported experiencing climate anxiety as a broader, future-oriented emotional response to the climate crisis. However, this form of concern was described primarily in relation to everyday life and societal developments, rather than athletes’ own competitive sport participation. Several participants expressed worry or emotional burden when confronted with climate change through media coverage and public discourse: “*When you hear about new reports in the news… or climate catastrophes… you start thinking that something has to change and that you should reconsider your own behavior*” (I1) or “*I would definitely say I feel concerned about climate change in general*” (I5). Most athletes did not perceive climate anxiety as directly relevant to their sport: “*Not in relation to my competitive sport. I do not think climate change will affect my sport that much in the time I’m still competing*” (I2) or: “*In combination with competitive sport, not so much, I just have not thought about it that much*” (I6).

Even when climate-related risks for sport were acknowledged, these thoughts were often described as short-lived or actively avoided: “*The thought crosses your mind that extreme weather could happen more often, but I tend to push that thought away*” (I8). Overall, climate anxiety appeared to be compartmentalized: While present in athletes’ everyday reflections, it was largely decoupled from their sport-specific experiences, suggesting a functional distancing that may help protect sport-related focus and performance.

A recurring pattern was the coexistence of negative affect related to ecological sustainability and positive emotions associated with sport participation. Athletes described how feelings of guilt or concern were often outweighed by enjoyment, motivation, and passion for their sport: “*There is some guilt, of course… but the joy and anticipation for competitions is stronger than that feeling*” (I4) or: “*The sport makes me very happy…but also feeling guilty and maybe a little bit ashamed*” (I6).

In addition, some athletes described positive emotions such as empowerment and hope, particularly when being involved in ecological sustainability initiatives or observing improvements:

*I feel sad about the thought that this sport is under a threat. I feel sad about having to like every time I come to a competition, and there’s no snow outside of the track, I feel sadness… But at the same time, I feel empowered when I talk to people who do, and when I’m part of this ambassador program, because I think we have been doing a lot of great things there* (I9).

All in all, athletes’ affective responses to ecological sustainability were characterized by a domain-specific pattern. While negative emotions such as guilt, sadness, frustration, and occasional anger were primarily related to environmental concerns, positive emotions such as enjoyment and passion were linked to sport participation. This appears central to how athletes maintain engagement and performance despite awareness of ecological sustainability challenges.

### 4.4 Self-regulation and coping

Self-regulation and coping responses were closely intertwined in athletes’ statements and served primarily to manage ecological sustainability-related cognition and affect in ways that protected sporting performance. Athletes described a range of coping strategies. A common pattern was cognitive disengagement during training and competition, with athletes intentionally downregulating ecological sustainability-related thoughts to maintain performance focus or not thinking about it at all: “*I just switch my head off. It does not really affect me in that moment*” (I2) or regaining the focus quickly on task relevant aspects: “*I think about it briefly… Then I focus back on the competition and the rest*” (I8). Sport itself was often framed as a regulatory resource, helping athletes cope with overwhelming emotions: “*When I’m competing, I’m competing. When I’m training, I’m training. And I think it’s actually also very good for the mental health to be training and to be competing*” (I9).

Outside of training and competition, several athletes reported engaging more actively with ecological sustainability-related issues through information-seeking and reflection. They described looking up alternatives, “*then I start thinking about what I could change myself and look for better options*” (I1), educating themselves “*to inform myself, what I could improve*” (I2), and thinking about how they could adjust their behavior “*it’s my responsibility that I think about what I could change*” (I7).

Coping was frequently described as socially embedded, with athletes emphasizing the importance of talking to others to process emotions, gain reassurance, and exchange knowledge. These social interactions were sometimes experienced as motivating and empowering: “*I talked a lot with my friends and family about what is bothering me*” (I6) or “*talking to others who think similarly gives me support*” (I5). Moreover, athletes at higher competition levels seek partnerships or use social media to inspire others and engage in projects: “*I do a lot of posting on social media. So recently I’ve had some collaborations with people that I find very inspiring. I’ve had partnerships with brands where we try to push out a message*” (I9).

Several athletes described attempts to compensate for the environmentally harmful aspects of their sport by acting more ecologically sustainable in other life domains: “*I try to compensate for the non-ecological aspects of my sport through other areas of my life*” (I4). At the same time, athletes differentiated between issues they perceived as controllable and those seen as structurally constrained by the organization of competitive sport. When perceived agency was low, athletes tended to disengage cognitively and emotionally: “*If it’s something I cannot really influence… I try not to think about it too much… because it’s exhausting*” (I7).

Taken together, the findings suggest that athletes regulate ecological sustainability-related cognition and affect through distancing, social processing, information-seeking, and compensatory actions. While some engaged with ecological concerns outside sport, many downregulated or compartmentalized these concerns during training and competition to preserve focus and performance.

### 4.5 Athletic performance

Most athletes reported that ecological sustainability-related cognition and affect were largely absent during their performance, particularly during competition. A dominant pattern was deliberate compartmentalization: Athletes described “switching off” ecological sustainability-related thoughts once performance was required: “*It does not affect my performance. It’s just a brief thought, and as soon as the game continues, it’s gone*” (I1). Especially athletes competing at a higher level cannot accept that these thoughts mitigate their performance: “*When I’m racing, I have to do it with 100%. Otherwise, you might as well not race at all*” (I2) and: “*When I’m competing, I’m competing; when I’m training, I’m training*” (I9). This absence was often explained through strong attentional focus and self-regulation as outlined beforehand, with athletes emphasizing their ability to suppress distracting thoughts: “*In the moment when I have to perform… I really try to switch my head off*” (I6).

Some athletes acknowledged that cognitions and affect were present in situations before or after the competition, particularly when ecological sustainability-related constraints interfered with preparation or recovery. Travel conditions were a recurring example, with long train journeys perceived as more exhausting than flights and thus potentially performance-relevant: “*When we were told we had to take the train instead of flying, we were quite frustrated, because we knew our performance the next day would not be optimal*” (I7) or were present right before practice: “*If I had dealt with it shortly beforehand, then maybe*” (I5) or during practice: “*In training (…) if the day was already going badly and the session did not go well, these emotions sometimes got me down a bit*” (I7). Similarly, disruptions caused by climate change and weather conditions were described as affecting training quality and competitive readiness: “*Some races had to be cancelled or postponed because of the weather. I had to do my final preparation by running instead of skiing, which is not optimal at all*” (I9) or: “*Artificial snow behaves very differently, and I’ve been unlucky with equipment not fitting the conditions*” (I9).

A few athletes also reflected that prolonged cognitive or emotional engagement with environmental concerns could affect performance via impaired recovery: “*If I keep thinking about it for a long time and cannot fall asleep, that would definitely affect my performance, because recovery is so important*” (I6). Interestingly, isolated emotional activation (e.g., anger about littering) was described as momentarily increasing their intensity, though athletes did not perceive this as performance-enhancing: “*Sometimes I run faster …because I’m angry, but that’s not really a smart decision*” (I8) or “*Yes, it actually makes me faster*” (I2).

Overall, sustainability-related cognition and affect were largely absent during the course of the sport competition, but were perceived as affecting sporting performance in periods of preparation and recovery. Thus, they were at work through structural conditions (e.g., travel, cancellations, altered training conditions) or cumulative mental load rather than through immediate in-competition distraction.

## 5 Discussion

The aim of the present study was twofold: First, to examine how competitive athletes cognitively and affectively engage with ecological sustainability within their sport, and second, to examine how athletes perceive ecological sustainability-related cognition and affect in relation to their sporting performance. Taken together, the findings suggest that ecological sustainability-related cognition and affect in competitive sport emerge from the interaction between athletes’ pro-environmental values and the structural demands of competitive sport. This interaction shaped athletes’ experiences of ecological sustainability and their perceived impacts on sporting performance, providing the basis for the individual and structural implications discussed below.

With regard to the first research aim, the results indicate that participants frequently expressed pro-environmental attitudes, environmental concern, and awareness of ecological sustainability across multiple behavioral domains. This interpretation is supported by athletes’ repeated reflections on the importance of environmental protection, sustainability-related challenges within competitive sport, feelings of responsibility for the environmental consequences of sport participation, and descriptions of efforts to adopt or promote more sustainable practices. In line with conceptualizations of pro-environmental behavior (Diekmann and Preisendörfer, 2003), athletes’ ecological sustainability-related cognition covered the domains of transport and mobility; consumption; waste and recycling; and energy and water saving. Among these, transport and mobility-related issues emerged as the most prominent categories. This finding is in line with earlier research, given that travel is one of the largest contributors to sport-related carbon emissions (Wilby et al., 2023) and is particularly visible in athletes’ everyday routines (IBU, 2026). The prominence of transport- and mobility-related cognition thus reflects both the objective environmental impact of sport-related mobility and athletes’ subjective awareness of it.

Despite these pronounced pro-environmental attitudes, athletes described difficulties translating their values into consistent pro-environmental behavior within their sporting context. This discrepancy reflects an environmental value-action gap (Blake, 1999). The present findings highlight that this gap is particularly pronounced in competitive sport due to structural and institutional constraints. Athletes reported limited autonomy in ecological sustainability-relevant domains, such as travel mode, competition schedules, equipment use, and sponsorship obligations. Several athletes reported compensating for the ecological impact of their sport by acting more ecologically sustainable in other life domains. This notion mirrors research on attitude-behavior inconsistencies, showing that value-action conflicts are often managed through behavioral compensation or cognitive rationalization (Lavergne and Pelletier, 2015). While cross-domain compensation may restore moral consistency under structural constraints, it risks normalizing ecologically unsustainable practices if it acts as a substitute for structural change. These findings underscore that ecological sustainability-related behavior in sport must be understood within broader organizational and institutional frameworks, not solely as a matter of individual motivation. The present findings also show parallels to climate action delay discourses identified by Lamb et al. Lamb et al. (2020) and recently examined in elite sport by Stegmann and Suter Stegmann and Suter (2026). Participants frequently described their limited influence over sustainability-relevant decisions, resembling narratives that redirect responsibility or portray the sport system as predetermined. At the same time, our findings support Stegmann and Suter’s (2026) argument that such narratives may not merely function as justifications for inaction, but may also reflect genuinely perceived limitations in agency within highly structured sport environments. This suggests that climate action delay in competitive sport may be driven not only by discursive strategies but also by athletes’ lived experiences of structural constraints.

In addition to cognitive engagement, athletes reported a range of climate change and ecological sustainability-related affect, including guilt, frustration, anger, sadness, and, in some cases, feelings of empowerment. These emotional responses often emerged in relation to perceived discrepancies between personal values and actual behavior, as well as in response to witnessing environmentally harmful practices by others. This pattern can be understood through cognitive dissonance theory (Festinger, 1957), which posits that inconsistencies between personal values and behavior elicit psychological discomfort and negative affect. The feelings of guilt, frustration, and anger reported by athletes can thus be interpreted as affective manifestations of such dissonance within the structurally constrained context of competitive sport. Moreover, this pattern aligns with theoretical accounts highlighting the emotional dimension of climate change (Böhm et al., 2023; Schwarz et al., 2026). Athletes also reported positive affective responses to ecological sustainability initiatives in sport (e.g., reduced plastic use at events), suggesting that institutional measures may foster both pro-environmental behavior and positive emotions (Böhm et al., 2023).

A notable finding concerns climate anxiety: While several athletes reported climate-related worries and distress in their everyday lives, these concerns were rarely perceived as directly threatening their own sport. Climate anxiety thus appeared more salient in a general life context than in the immediate sporting context. This suggests a psychological compartmentalization (Thomas et al., 2013), whereby climate-related concerns are acknowledged but decoupled from the domain of sport. Similar patterns have been observed among adolescents and young adults, who compartmentalized concerns about the climate crisis from activities that provide positive affect (Shrestha et al., 2025). In the present study, competitive sport appeared to serve a comparable function. These mechanisms can be seen as self-protective strategies against climate change threats (Wullenkord and Reese, 2021). This pattern adds nuance to existing research on climate anxiety by demonstrating that the emotional impact of climate-related concerns may vary across situational contexts.

However, athletes should not be understood solely as passive recipients of structurally constrained sport systems. Several participants explicitly acknowledged their own contribution to environmentally consequential practices through travel, equipment use, and participation in competition structures. At the same time, these practices were often perceived as necessary to meet performance demands, which is in line with previous research (Wågan and Wilson, 2025). For example, some athletes preferred air travel over more environmentally friendly alternatives because longer train journeys were viewed as detrimental to preparation and recovery. Similar tensions emerged regarding sport equipment and apparel. While athletes recognized the environmental implications of frequent equipment replacement and clothing consumption, many described these practices as driven by sponsorship arrangements, team provisions, or performance requirements but sometimes also as personal preference. Thus, athletes appeared both as beneficiaries of and participants in environmentally consequential sport systems, while simultaneously perceiving limited influence over many sustainability-relevant decisions. The perceived costs of foregoing air travel were often considered too high in relation to expected performance consequences. Prior research highlighted that environmental attitudes only promote environmental actions in low-cost situations (Diekmann and Preisendörfer, 2003).

Addressing the second research aim, the findings indicate that athletes perceived ecological sustainability-related cognition and affect to be largely absent during training and competition, while describing context-dependent influences on preparation, travel, recovery, and performance readiness. Participants consistently described a strong ability to “switch off” ecological sustainability-related thoughts and emotions. Prior research has shown that athletes report high levels of attentional focus and task-oriented engagement in competitive situations to shield themselves from off-field concerns, thereby favoring performance-relevant cues (Bracco et al., 2025; Mann et al., 2007). The absence during performance can be understood through the coping strategies athletes employ. Cognitive distancing, selective attention, and deliberate suppression of ecological sustainability-related thoughts function as short-term regulatory strategies that protect performance (Nuetzel, 2023). However, some athletes reported that when directly exposed to ecologically unsustainable behavior by others, they reacted by increasing effort (e.g., running or cycling faster). Participants perceived that these situations disrupted their attentional focus and race strategy. This interpretation aligns with research suggesting that task-irrelevant thoughts may impair performance under competitive conditions (Lautenbach et al., 2025; Li et al., 2024).

At the same time, the findings point toward context-dependent pathways through which ecological sustainability-related cognition and affect may affect performance. These concerns were described as becoming more salient outside competition phases, for instance, during travel, recovery, or periods of high mental load. In these phases, prolonged rumination, negative affect, or climate-related distress occasionally interfered with sleep, recovery, and general well-being, which are determinants of performance readiness (Charest and Grandner, 2020; Schorb et al., 2025). This pattern is consistent with models of self-regulation and mental resource depletion, which provide one possible theoretical explanation for the perceived influence of ecological sustainability-related concerns on recovery and performance maintenance (Englert, 2016; Hagger et al., 2010). Thus, while performance is often protected in the moment, cumulative mental load may exert more subtle long-term effects.

Moreover, athletes reported tensions arising from ecological sustainability-oriented decisions themselves. Choosing more environmentally friendly travel options was sometimes perceived as high-cost situations (Diekmann and Preisendörfer, 2003), increasing travel time, fatigue, or logistical strain, which could impair recovery and performance readiness. This finding highlights a structural dilemma: Ecological sustainability measures may conflict with performance optimization within current competitive systems. These tensions may foster negative affect or conflicting emotional responses toward ecological sustainability initiatives when they are experienced as imposed burdens rather than enabling conditions (van Gent et al., 2024).

Finally, sport itself emerged as an important emotional and cognitive “vent”: Training and competition, particularly in natural environments, were described as providing relief from overwhelming climate-related concerns, supporting mental well-being, and regulating emotions (Firnhaber et al., 2025). This aligns with research on the restorative effects of physical activity and nature exposure on mood, cognition, and ego depletion (Beute and Kort, 2014; Fu et al., 2025). Thus, sport can serve both as a domain of ecological tension and as a coping resource for dealing with cognitive and affective demands.

The findings have implications for athletes, coaches, sport organizations, and event organizers. Athletes experienced a pronounced environmental value-action gap, largely driven by institutional constraints such as competition calendars, travel requirements, qualification systems, and sponsorship regulations. This indicates that ecological sustainability cannot be addressed solely at the individual level. Federations and governing bodies should assume greater structural responsibility by integrating ecological sustainability criteria into scheduling, competition formats, and travel planning (e.g., regional clustering of events, coordinated travel). Reducing these barriers may lower environmental impact and alleviate psychological strain arising from ecological sustainability-related tensions. At the same time, federations are embedded in broader institutional and economic dependencies. For instance, in Germany, biathlon events in Ruhpolding and Oberhof are scheduled around the Bundesliga winter break, illustrating that sport calendars are shaped by cross-sector interdependencies. Ecological sustainability transitions in sport require systemic coordination rather than isolated, sport-specific measures. However, athletes should not only be considered recipients of structural change but also contributors to sustainability transitions in sport. The present findings suggest that engagement is more likely when athletes perceive meaningful opportunities to influence their sport environment rather than being confronted solely with individual responsibility for systemic problems. Consistent with recent work on climate action delay in elite sport (Stegmann and Suter, 2026), the present findings suggest that sustainability transitions should address both structural conditions and athletes’ cognitive and emotional experiences. Involving athletes in sustainability-related decision-making processes, developing sport-specific sustainability strategies together with federations, and communicating realistic pathways for change may strengthen athletes’ sense of agency and foster collective rather than purely individual responsibility for ecological sustainability.

Athletes noticed and valued visible ecological sustainability measures at venues (e.g., waste and recycling management, energy-saving practices). Even small, tangible changes were perceived positively and sometimes elicited empowerment. Event organizers can build on this by implementing and clearly communicating concrete ecological sustainability practices, which may support both ecological goals and athletes’ perceived value coherence. At the same time, such communication should avoid merely symbolic actions or exaggerated environmental claims, as these may be perceived as greenwashing and undermine the credibility of sustainability efforts (Paruzel et al., 2026; Kraaij et al., 2026).

Lastly, athletes also reported suppressing ecological sustainability-related thoughts and emotions in performance contexts to maintain focus. While functional in the short term, this may contribute to cumulative mental load. Coaches and sport psychologists could provide structured opportunities for athletes to reflect on ecological sustainability-related tensions. Such reflection should not aim to diminish environmental concern, but rather to facilitate constructive emotional processing, strengthen perceived agency, attenuate negative influences on performance, and help athletes identify opportunities for individual and collective engagement where meaningful. These opportunities appear particularly relevant given emerging evidence that climate-related worry can motivate environmental engagement when accompanied by hope, positive affect, and opportunities for action (Kleres and Wettergren, 2017; Bechtoldt and Schermelleh-Engel, 2024). Such support may, therefore, contribute not only to athlete well-being and performance, but also to broader sustainability transitions within sport by fostering informed dialogue, collective responsibility, and engagement with systemic change.

## 6 Conclusion

This study examined how competitive athletes cognitively and affectively engage with ecological sustainability and how these cognitions and affects shape sporting performance. Athletes expressed strong pro-environmental attitudes but experienced pronounced tensions with the structural demands of competitive sport, particularly regarding transport and mobility. These discrepancies elicited diverse cognitive and affective responses, including rumination, guilt, sadness, and, at times, empowerment through engagement. Addressing ecological sustainability in competitive sport requires coordinated action across multiple levels. While many sustainability-relevant decisions are structurally determined by federations, organizers, and sponsors, athletes can contribute to sustainability transitions through advocacy, participation in sustainability initiatives, and engagement in discussions about more sustainable sport systems. Importantly, the present findings do not suggest that sustainability-related concerns or negative emotions should be eliminated. Rather, in line with research showing that emotions such as climate worry, guilt, or fear can motivate environmental engagement, agency, and opportunities for collective action (Kleres and Wettergren, 2017; Bechtoldt and Schermelleh-Engel, 2024), creating meaningful opportunities for athletes to engage in sustainability while reducing unnecessary structural barriers may help translate environmental concern into constructive action.

This study contributes to environmental psychology by extending research on ecological sustainability-related cognition and affect to the under-researched context of competitive sport. The findings illustrate how pro-environmental attitudes coexist with institutional constraints that foster value-action gaps, underscoring the role of situational and organizational factors in environmental engagement. By linking ecological sustainability-related cognition and affect to self-regulation and coping in high-performance settings, the study bridges environmental and sport psychology and highlights how environmental concerns interact with performance demands.

Several limitations should be considered, which provide directions for future research. First, the reliance on qualitative interviews may limit introspective access. Diary studies could more systematically capture the intensity and situational variability of ecological sustainability-related cognition and affect, including variations across competitive situations or phases of the season, which may shape the salience of sustainability-related concerns (e.g., off-season vs. competition periods). Additionally, the recruitment materials explicitly referred to ecological sustainability in sport. As a result, self-selection bias cannot be ruled out, as athletes with stronger environmental interests or greater engagement with sustainability issues may have been more likely to participate. However, ethics protocols request that the actual purpose of the study is revealed to (potential) participants.

The present findings provide an empirical basis for the development of context-specific quantitative measures (e.g., questionnaires), enabling theory testing, comparability across studies, and the examination of performance-related outcomes at scale. In addition, future research could systematically compare contexts (e.g., elite vs. amateur, individual vs. team, indoor vs. outdoor or nature-based sports) and include more diverse athlete groups, particularly regarding age. The relatively young athletes in this study may differ from older athletes in how they experience ecological sustainability issues. Furthermore, the rather small and heterogeneous sample in terms of sport type and competitive level may have influenced the findings. Although thematic saturation was reached with respect to the present research questions, additional sport-specific perspectives may emerge in samples with broader representation across sport types and a higher representation of elite-level athletes. For example, athletes competing at higher levels often face greater travel demands, sponsorship obligations, and performance pressures, which may intensify sustainability-related tensions and value-action conflicts. Conversely, athletes competing at lower levels may be more directly affected by climate-related disruptions, as they and their sport organizations often have fewer financial and organizational resources to adapt to environmental challenges (Orr and Inoue, 2019). Similarly, sustainability-related experiences may differ across sports depending on climate change exposure, dependence on natural environments for sport practice and competitions, and the role of environmental concerns within athletes’ sport identities. Moreover, incorporating perspectives from other stakeholders in sport (e.g., coaches or sport psychologists) may help to better capture structural constraints and coping processes. Finally, the study focused on perceived rather than objective performance outcomes. Future research should incorporate objective performance indicators (e.g., physiological data or competition results) and experimental designs.

## Data Availability

The raw data supporting the conclusions of this article will be made available by the authors, without undue reservation.

## Footnotes

1 These research questions deviate slightly from the preregistered version. In the preregistration, cognitive and affective processes were addressed separately. For clarity and coherence, they are integrated here into a combined framework reflecting their interrelated nature. The focus on perceived relevance remains consistent with the original research intent.

2
https://osf.io/tmej9/files/sryvq
