# Correction: “I know it's not good, but you want to perform at 100%”: cognitive-affective responses to ecological sustainability and performance in sport competitions

**DOI:** 10.3389/fpsyg.2026.1995457

**Published:** 2026-09-29

**Authors:** Christian Kraft, Rabea Bödding, Maximilian Gottwill, Christian Zierke, Agnieszka Paruzel, Günter W. Maier, Pamela Wicker

**Affiliations:** 1 Department of Sports Science, Bielefeld University, Bielefeld, Germany; 2 Department of Psychology, Bielefeld University, Bielefeld, Germany

**Keywords:** athletes, climate change, emotions, environmental sustainability, sport performance

There was a reference error in Figure 2. The reference for Russell (1980) was erroneously written as Dorris et al. (2012). The reference should be Russell, J. A. (1980). A circumplex model of affect. *J. Pers. Soc. Psychol*. 39, 1161–1178. doi: 10.1037/h0077714.

The content of Figure 2 itself is correct; only the corresponding source was inadvertently not updated during the revision of the manuscript.

The original version of this article has been updated.

