# Supplementary material for: “I know it’s not good, but you want to perform at 100%”: cognitive-affective responses to ecological sustainability and performance in sport competitions
Source: Front Psychol. 2026 Aug 21;17:1880290. doi: 10.3389/fpsyg.2026.1880290 (PMC13541440; doi:10.3389/fpsyg.2026.1880290)
Supplement: Supplementary file 1 [file Supplementary_file_1.docx]

Supplementary Material

Interview guide

**Introduction:**

Thank you for taking the time for this interview.
In this conversation, we’re interested in your **personal experiences, thoughts, and perceptions** related to **ecological sustainability and environmental issues** in the context of your competitive sport. We’d like to understand how you encounter these topics in your everyday sporting practice.

There are no "right" or "wrong" answers – we're interested in your **individual perspective**. You also don't need to answer immediately; take your time if needed.

The conversation may also touch on **thoughts or emotions that affect your competitive performance** – we’d like to better understand that, too.

The interview will take about **30 minutes**. If you feel uncomfortable with any question, you don’t have to answer it. You can end the interview at any time without giving a reason. You’ll find all relevant information in the **study information sheet** and **privacy policy**.

In this interview, we're particularly interested in the **presence of thoughts and feelings regarding ecological sustainability** and how these may influence your competitive sporting behavior and performance.

Before we begin, do you have any questions about the information sheet, the privacy policy, or the consent form?

If you're ready and agree, I’ll now start the audio recording.

| **No.** | **Guiding Questions** |
| --- | --- |
| 1 | How old are you? |
| 2 | What gender do you most identify with? |
| 3 | Which competitive sport do you practice, and at what level? |
| 4 | How long have you been involved in this competitive sport? |
| 5 | How would you describe your personal attitude toward ecological sustainability or environmental protection – in general? |
| 6 | In what ways does ecological sustainability play a role in your everyday competitive sport practice? Think of areas like travel/mobility for competitions, purchase/use of equipment, energy or water use (e.g., showers), or waste and recycling in your sport environment. |
| 7 | What thoughts do you have about ecological sustainability or environmental impacts related to your competitive sport? |
| 8 | What kind of feelings do you experience when thinking about ecological sustainability in your sport or when confronted with the issue? |
| 9 | What do you do when you notice that you're emotionally or mentally occupied with ecological sustainability issues? |
| 10 | Is there anything else related to this topic that you feel is important and we haven’t addressed yet? |

**References**

Birrer, D., Röthlin, P., & Morgan, G. (2012). Mindfulness to enhance athletic performance: Theoretical considerations and possible impact mechanisms. *Mindfulness, 3*, 235–246.

Chen, D., Nien, J., Geng, X., Yu, J. X., Singhnoy, C., & Chang, Y. (2025). Relationship between ruminative dispositions and perceived sports performance in young elite athletes in Hong Kong: the role of problem-oriented coping strategies. *Frontiers in Sports and Active Living, 7*,1513277.

Clayton, S. (2020). Climate anxiety: Psychological responses to climate change. *Journal of Anxiety Disorders, 74*, 102263.

Corrêa, C. G. L. (2024). The relation between affection and cognition: Theoretical perspectives. *Psicologia Escolar e Educacional, 28*, e257346.

Gaudreau, P., Blondin, J. P., & Lapierre, A. (2002). Athletes' coping during a competition: relationship of coping strategies with positive affect, negative affect, and performance–goal discrepancy. *Psychology of Sport and Exercise, 3*(2), 125-150.

Kröhler, A., & Berti, S. (2019). Taking action or thinking about it? State orientation and rumination are correlated in athletes. *Frontiers in Psychology, 10*, 576.

Langseth, T. and Vyff, A. (2021). Cultural dissonance: surfers' environmental attitudes and actions. *Frontiers in Sports and Active Living, 3*, 695048.

Lazarus, R. S. (1984). Stress, appraisal, and coping. Springer.

Lopes, G. C. D. (2024). The Influence of psychology on sports performance: emotional and cognitive factors. *South Florida Journal of Development, 5*(11), e4683.

Michel-Kröhler, A., Krys, S., & Berti, S. (2024). Rumination in the context of individual goal achievement: A cross-sectional study using a multisport sample. *Journal of Applied Sport and Exercise Psychology, 31*(2), 49–57.

Urquijo, I., Borrajo, E., Gonzalez-Santamaria, X., & Alcaraz, S. (2023). Antecedents of perceived stress in trail runners: do emotion regulation and negative self-talk matter? *International Journal of Sport and Exercise Psychology, 22*(8), 1969–1984.
